# Complete Genome Sequence of Paenibacillus polymyxa DSM 365, a Soil Bacterium of Agricultural and Industrial Importance

**DOI:** 10.1128/mra.00329-22

**Published:** 2022-05-16

**Authors:** Santosh Kumar, Victor C. Ujor

**Affiliations:** a Metabolic Engineering and Fermentation Science Group, Department of Food Science, University of Wisconsin-Madison, Madison, Wisconsin, USA; Loyola University Chicago

## Abstract

We report the complete genome sequence of Paenibacillus polymyxa DSM 365. The genome consists of a 5,788,318-bp chromosome, with a GC content of 45.48%. Annotation of the genome revealed a total of 5,246 genes (average length, 943 bp). Gene function analysis indicated the ability to fix nitrogen (N_2_) and to produce value-added chemicals.

## ANNOUNCEMENT

Paenibacillus polymyxa DSM 365 is a Gram-positive plant growth-promoting rhizobacterium (1) with capabilities for N_2_ fixation and production of antimicrobials and commercially relevant chemicals (1–7). P. polymyxa DSM 365 was procured from the German Collection of Microorganisms and Cell Cultures GmbH (Leibniz Institute DSMZ GmbH). To isolate DNA, cultures were grown overnight in tryptic soy broth at 30 °C and 200 rpm. Genomic DNA was extracted using the Wizard high-molecular-weight (HMW) DNA extraction kit (Promega, Madison, WI, USA). Library preparation and sequencing were conducted by Novogene Inc. (Sacramento, CA) using the Illumina NovaSeq 6000 platform. To prepare the library for sequencing, genomic DNA was randomly sheared into short fragments. The fragments were end repaired, adenine tailed, and ligated with Illumina adapters. The quantified libraries (350-bp size) were pooled and sequenced to produce 6 Mb of paired-end 150-bp reads (1,800 Mb of raw data). In order to ensure accuracy and reliability, the reads were filtered using readfq software (v.10) (8) with default parameters to screen out low-quality data. The resulting 5,286,666 reads were assembled using SOAPdenovo (v.2.04) (9, 10), SPAdes (v.3.10.0) (11), and ABySS (v.1.3.7) (12) assembly software with default settings. Before assembly, the genome size was estimated by k-mer analysis (9). Assembly results from the three software tools were integrated with the Contig Integrator for Sequence Assembly (CISA) database (13). GapCloser (v.1.12) (14) was used to fill the gaps in the preliminary assembly. Fragments of less than 500 bp were filtered out, and the final result was counted for gene prediction. The assembly data revealed a total of 5,788,318 bp (*N*_50_, 357,841 bp) in 47 scaffolds, with a GC content of 45.48% and an average read coverage of 291×.

GeneMarkS (v.4.10) (15) was used to identify coding genes, and noncoding RNAs were scanned using tRNAscan-SE, RNAmmer, and BLAST with the Rfam database (16–18). Interspersed repeats were predicted using RepeatMasker (v.4.0.9) (19), tandem repeats were predicted using Tandem Repeats Finder (v.4.09) (20), and clustered regularly interspaced short palindromic repeat (CRISPR) sequences were predicted using CRISPRFinder (v.2.0.3) (21).

The whole-genome sequence of P. polymyxa DSM 365 was submitted to the National Center for Biotechnology Information (NCBI) database using the Prokaryotic Genome Annotation Pipeline (PGAP) (v.6.0) (22). Homology-based gene prediction detected a total of 5,246 genes (85.43% of the total genome), with 4,966 protein coding sequences (CDSs), 156 RNA genes (tRNA, 104 genes; 5S rRNA, 13 genes; 16S rRNA, 18 genes; 23S rRNA, 17 genes; 4 ncRNA genes), and 104 pseudogenes. All of the protein sequences were aligned to the genome sequences using BLAST, and then GeneWise (23) was used to predict gene structure-based reliable alignments (E value of <1*e*^−5^). Coding genes were predicted by Augustus (v.2.7) (24) with homologous evidence. Several genes encoding enzymes involved in carbohydrate metabolism (e.g., rhamnogalacturonan lyase, cellulase, and cellobiohydrolase), nitrogen fixation (*nif* operon), sporulation, acetoin utilization, biosynthesis of siderophores, polyketides, exopolysaccharides, and butanediol were detected.

### Data availability.

The annotated genome sequence of P. polymyxa DSM 365 has been deposited in GenBank under the BioProject accession number PRJNA809744, the BioSample accession number SAMN26200526, and the Sequence Read Archive (SRA) accession number SRR18173204. The whole-genome shotgun project has been deposited in DDBJ/ENA/GenBank under the accession number JAKVDC010000000.
